# Diversity begets diversity: host expansions and the diversification of plant-feeding insects

**DOI:** 10.1186/1471-2148-6-4

**Published:** 2006-01-18

**Authors:** Niklas Janz, Sören Nylin, Niklas Wahlberg

**Affiliations:** 1 Department of Zoology, Stockholm University, 106 91 Stockholm, Sweden

## Abstract

**Background:**

Plant-feeding insects make up a large part of earth's total biodiversity. While it has been shown that herbivory has repeatedly led to increased diversification rates in insects, there has been no compelling explanation for how plant-feeding has promoted speciation rates. There is a growing awareness that ecological factors can lead to rapid diversification and, as one of the most prominent features of most insect-plant interactions, specialization onto a diverse resource has often been assumed to be the main process behind this diversification. However, specialization is mainly a pruning process, and is not able to actually generate diversity by itself. Here we investigate the role of host colonizations in generating insect diversity, by testing if insect speciation rate is correlated with resource diversity.

**Results:**

By applying a variant of independent contrast analysis, specially tailored for use on questions of species richness (MacroCAIC), we show that species richness is strongly correlated with diversity of host use in the butterfly family Nymphalidae. Furthermore, by comparing the results from reciprocal sister group selection, where sister groups were selected either on the basis of diversity of host use or species richness, we find that it is likely that diversity of host use is driving species richness, rather than vice versa.

**Conclusion:**

We conclude that resource diversity is correlated with species richness in the Nymphalidae and suggest a scenario based on recurring oscillations between host expansions – the incorporation of new plants into the repertoire – and specialization, as an important driving force behind the diversification of plant-feeding insects.

## Background

The biodiversity crisis calls for a better understanding not only of the reasons for loss of diversity, but also for the processes that generate diversity. Plant-feeding insects are remarkably species-rich, making up at least one-quarter of all described species, so explaining the mechanisms behind the diversification of these groups will go a long way towards understanding global biodiversity [1,2]. The possible link between insect diversification and feeding on plants was made already by Ehrlich and Raven [3] in their seminal paper on the coevolution between butterflies and plants. Since then, it has been clearly demonstrated that herbivory has repeatedly led to rapid diversification of insects, but the mechanisms behind this diversification still remain uncertain [4,5]. Compared with alternative resources, plants are characterized by both high availability and high diversity. Insect diversification rates could conceivably be influenced by both resource abundance (decreased competition) and diversity (larger number of potential niches), but these hypotheses have so far not been tested with phylogenetic methods.

It has become clear that ecological factors can cause rapid speciation and evolutionary divergence [6]. For plant-feeding insects, the widespread specialization on a diverse resource has been seen as a likely ecological mechanism behind the rapid diversification [7-11]. The word specialization can refer to both a state and a process. The specialization process will give rise to an increasingly specialized state, by decreasing the number of plants used as hosts. It is as a process that specialization can influence speciation rates, and to emphasize this aspect we use the term, even when referring to states, in a relative sense; an insect that uses two plant species as hosts is for instance less specialized than an insect that uses one, but more specialized than an insect that uses three [c.f. [12]].

There are at least two ways that specialization can promote speciation rates: either by a genetic linking between resource use and mate choice, which could create "host races" with an increasing genetic differentiation [10,13-15], or because a resource specialist's host will tend to be more patchily distributed and thus increase the likelihood of differentiation among populations [11,16].

Both these mechanisms appear valid [10,11] but they only provide a mechanism for part of the process; the actual breaking up of an existing coherent species into distinct daughter species. Specialization is essentially a pruning process, preserving certain existing interactions at the expense of others. This can cause divergence, if there is a structuring factor, such as geographic heterogeneity or resource-based assortative mating, and if different resources are favored in different subsets of the species. However, by its own action, the process would soon run out of "fuel" – the variation in host use that drives the process. Once a species has reached a truly specialized state, further specialization is impossible. Therefore, we also need to incorporate a process that is inherently diversifying. Something must cause the original species to have a widespread distribution – or to have several host species to form host races on – in the first place.

If indeed diversification was only driven by specialization, we would see a never-ending drive towards increasing specificity and ultimately all further diversification could only be accomplished by cospeciation with the host. With the increasing availability of phylogenetic information and better understanding of the coevolutionary process this "dead-end" view of specialization and the corresponding cospeciation scenario has been challenged. First, the increased likelihood of extinction will tend to counterbalance speciation rates in highly specialized lineages [17]. Moreover, most interactions, even the most specialized, are evolutionarily dynamic, where the possibility of generalization is always present [9,12,17-24]. Finally, despite many attempts, cospeciation has rarely been found among plant-feeding insects. Most studies have instead concluded that host colonizations and shifts are much more important processes behind the patterns of insect-plant associations [12,24-34]. Moreover, in many cases the patterns of host use cannot logically be attributed to cospeciation due to asynchrony in diversification events for the associated groups of plants and insects [27,30].

Colonization of novel host plants is an evolutionary process that is capable of generating new variation in host use, and could thus conceivably be the "missing fuel" in the engine of diversification. Even if there seem to be a general conservatism in host use among most groups of plant-feeding insects [3,27,35], there have also been studies which have seen a great deal of evolutionary flexibility in host use, with numerous colonizations and host shifts, sometimes even in ecological time [26,36-39]. More detailed phylogenetic studies have also revealed a more dynamic pattern of host use than the more large-scale assessments suggested, probably because many of the host colonizations seemed to involve a limited set of plant groups – "building blocks" of host plant range that can be combined in different ways [12,40-43]. Phylogenetic reconstructions within the butterfly tribe Nymphalini have suggested that more radical host shifts were more common during periods of host range expansion, and that this diversification of host use appeared to be connected to increased speciation rates [12,44], but the sample sizes were too small to draw any general conclusions.

If species richness among plant-feeding insects has been promoted by the diversification of the plant interaction, there should be a general correlation between host diversity and species richness. The main objective of this study is therefore to test if species richness is correlated with resource diversity in the butterfly family Nymphalidae, and to provide a plausible mechanism for this diversification. To test this hypothesis, we have performed an independent contrast analysis to look for a general phylogenetic correlation between diversity of host plants and species richness. To further address the question of causality we also performed reciprocal sister group analyses where either host diversity or species richness was used as a basis for sister group selection.

## Results and discussion

The correlation between diversity of host use and species richness was tested with the computer program MacroCAIC [45], which applies the method of independent contrasts to questions of species richness. The contrasts generated by MacroCAIC did not meet assumptions for regression analysis, but a Wilcoxon matched-pair signed-rank test on the independent contrasts showed a strong positive association between contrasts for host plant diversity and species richness (N = 204, z = 6.446, p << 0.001; Fig. 2). The test is correlational and causation could conceivably go both ways, but it clearly demonstrates that diversity of host use is correlated with host plant diversity.

**Figure 1:**
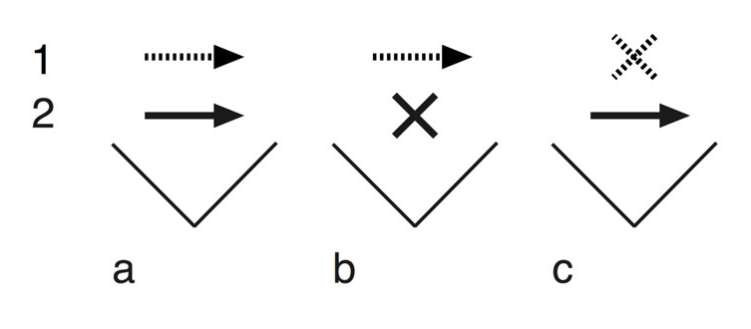
**Reciprocal sister group comparisons**. A schematic illustration of the application of reciprocal sister group comparisons to evaluate questions of causation for associated traits in situations where only correlational analyses are possible, but where the correlation is not absolute. **(a) **Trait 1 and 2 are positively correlated across the phylogeny, so that a difference in one trait is associated with a correlated difference in the other. **(b) **Provided that the correlation is not perfect, there will be situations where a difference in trait 1 is not associated with a correlated difference in trait 2, and **(c) **conversely, where a difference in trait 2 is not associated with a correlated difference in trait 1. This can be interpreted to mean that the traits, although statistically influenced by each other, sometimes evolve for external reasons. If (b) is more common than (c), trait 1 can sometimes change without an associated change in trait 2, while trait 2 rarely evolves without an associated change in trait 1. Hence, trait 2 does not necessarily follow the evolution of trait 1, but trait 1 appears to follow the evolution of trait 2.

**Figure 2:**
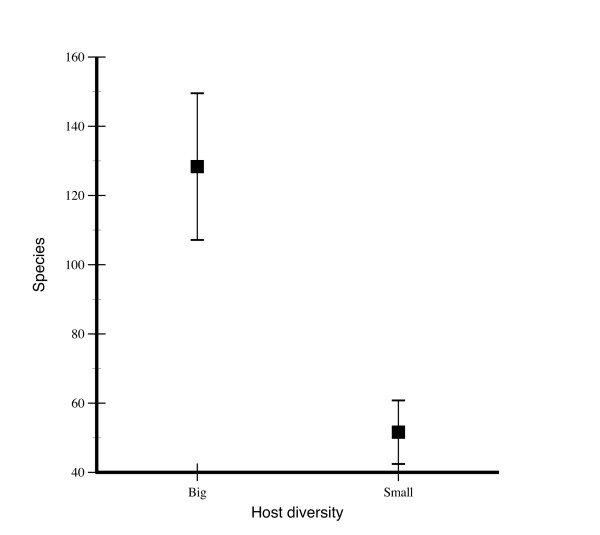
**Phylogenetically independent contrasts generated by MacroCAIC**. The figure shows species numbers of the clades connected by the independent contrasts in host plant diversity found by MacroCAIC. There is a positive correlation between host diversity and species richness; clades with a higher host diversity index also had significantly higher species numbers. Means ± SE.

**Figure 3:**
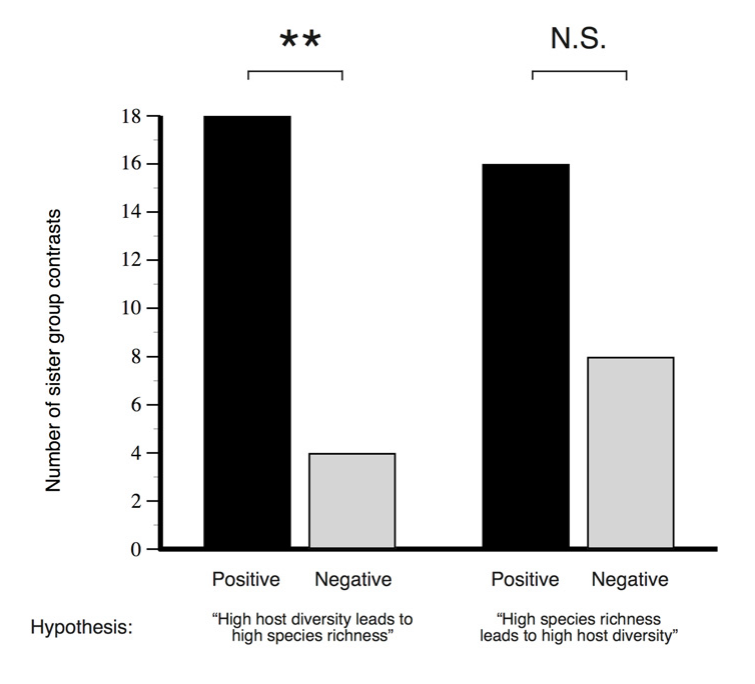
**Results from reciprocal sister group comparisons**. Results from the two reciprocal methods for sister group selection. The bars to the left show to what extent contrasts in host diversity is positively correlated with species richness, the bars to the right show to what extent contrasts in species richness is positively correlated with host diversity. When selecting sister groups on the basis of differences in host plant diversity the correlation was more pronounced than when using species richness as the basis for sister group selection. The different outcome of the two methods of sister pair selection suggests that while increases in species richness is not necessarily followed by higher host diversity, increased host diversity is predictably followed by increased species richness.

To address the question of causality we performed reciprocal sister group comparisons, where sister clades were chosen on the basis of either differences in host plant diversity or in species numbers (Fig. 1). There were 22 valid sister pairs in the phylogeny that differed in host diversity. Of these, 18 showed a positive correlation with species richness (Sign test, p = 0.004). By using the reciprocal method of pairing selection there were 24 pairs that differed in species richness and among these, 16 were positively correlated with host diversity (Sign test, p = 0.152). Hence, the correlation was more pronounced when using host plant diversity as a basis for sister group selection than when using species numbers (Fig. 3; Table 1 and 2). The different outcome of the two methods of sister pair selection suggests that host plant numbers do not automatically increase with increasing species numbers. This means that the relationship between host range and species richness is not absolute and that there must be cases where speciation events have apparently not been associated with increases in host diversity, which is hardly surprising. On the other hand, when there has been an increase in host diversity, this is almost always followed by an increase in species richness. Consequently, the data is more consistent with the hypothesis that it is host plant diversity that influences species numbers rather than vice versa.

**Table 1:** Sister groups differing in host diversity (HD). Sister groups were selected on the basis of differences in host diversity. HD 1 is the group with the highest host diversity and Richness 1 is the corresponding species richness of that group. Richness 2 shows the species richness of the group with the lower host diversity (HD 2).

Sister pairing	HD 1	HD 2	Richness 1	Richness 2
*Libythea-Libytheana*	2	1	7	4
*Lycorea-Anetia*	9	1	3	5
*Parantica-Ideopsis*	2	1	39	8
*Charaxes-Polyura*	247	9	189	21
*Actinote/Acraea-Pardopsis*	143	1	255	1
*Vagrans/Cupha-Phalanta*	9	6	9	5
*Cymothoe-Harma*	6	2	69	1
*Cyrestis-Chersonesia*	2	1	18	6
*Eunica-Sevenia*	6	1	40	15
*Colobura-Tigrida*	2	1	2	1
*Polygonia-Kaniska*	35	1	13	1
*Vanessa-Hypanartia*	243	1	19	14
*Hypolimnas-Precis*	40	1	23	15
*Yoma-Protogoniomorpha*	4	1	2	5
*Doleschallia-Kallima/Catacroptera/Mallika*	4	1	9	11
*Anthanassa/Telenassa/Eresia/Castilia-Tegosa*	9	1	57	14
*Hamadryas-Panacea/Batesia*	2	1	20	4
*Morpho-Antirrhea/Caerois*	16	4	32	13
*Eueides-Heliconius/Laparus/Neruda*	2	1	12	44
*Argynnis s.l.-Brenthis*	4	1	41	4
*Chlosyne-Microtia*	8	4	33	5
Rest of *Brassolini-Bia*	55	1	92	2
Positive contrasts	22 (100%)		18 (82%)	

**Table 2:** Sister groups differing in species richness. Sister groups were selected on the basis of differences in species richness. Richness 1 is the group with the highest richness and HD 1 is the corresponding host diversity index for that group. HD 2 shows the host diversity index for the group with the lower species richness (Richness 2).

Sister pairing	Richness 1	Richness 2	HD 1	HD 2
*Libythea-Libytheana*	7	4	2	1
*Anetia-Lycorea*	5	3	1	9
*Parantica-Ideopsis*	39	8	2	1
*Charaxes-Polyura*	189	21	247	9
*Actinote/Acraea-Pardopsis*	255	1	143	1
*Dione-Agraulis*	3	2	1	1
*Cupha-Vagrans*	8	1	4	4
*Cymothoe-Harma*	69	1	6	2
*Cyrestis-Chersonesia*	18	6	2	1
*Eunica-Sevenia*	40	15	6	1
*Colobura-Tigrida*	2	1	2	1
*Polygonia-Kaniska*	13	1	35	1
*Vanessa-Hypanartia*	19	14	243	1
*Symbrenthia-Mynes*	11	10	1	1
*Hypolimnas-Precis*	23	15	40	1
*Protogoniomorpha-Yoma*	5	2	1	4
*Oleria-Hyposcada*	50	8	1	1
*Heliconius/Laparus/Neruda-Eueides*	44	12	1	2
*Argynnis s.l.-Brenthis*	41	4	4	1
*Hamadryas-Panacea/Batesia*	20	4	2	1
*Anthanassa/Telenassa/Eresia/Castilia-Tegosa*	57	14	9	1
*Chlosyne-Microtia*	33	5	8	4
*Kallima-Catacroptera/Mallika*	9	2	1	1
Rest of *Brassolini-Bia*	92	2	55	1
Positive contrasts	24 (100%)		16 (67%)	

We have previously shown that diverse host plant use within the tribe Nymphalini was typically caused by ancestral polyphagy, which may or may not have secondarily evolved into more specialized interactions [12]. Virtually all documented host plant colonizations in that study led to host expansions, not to direct host shifts, and the evolutionary trend in this group was actually towards increased generalization rather than specialization. These butterflies appears to have been caught in a phase where a lowered host specificity allowed them to experiment with novel hosts, and to repeatedly reshuffle a common set of host plants, the building blocks of the host range. It appears likely that most lineages of plant-feeding insects pass through such phases of "evolutionary tinkering", where host use is expanded and diversified [c.f. [46,47]]. Indeed, in order to complete a host shift, a species must pass through a phase of expanded host range, where both the ancestral and novel plants are used. The length of this phase will vary, depending on selection pressure and ecological setting. There are examples where fitness on the ancestral host has decreased substantially over ecological time scales [37], but on the other hand, there is evidence that ancestral hosts can linger in the repertoire for several tens of millions of years [12,42].

Even if specialization is a pervasive pattern, the process is not irreversible and occasional episodes of host expansions could conceivably generate the necessary variation in host use to drive speciation. Expansions during shifts to novel hosts are also well documented on an ecological level [37,48,49]. The importance of such host expansions has probably been neglected because these phases are evolutionarily short-lived and will tend to evolve into more specialized interactions again over time [12,24], but despite their ephemeral nature they may play a key role in the diversification of plant-feeding insects.

Even if the actual "polyphagy event" may be lost in history due to secondary specialization, the combined present range of hosts within a taxon should be a relatively accurate reflection of the ancestral host range. Hence, even if polyphagy is a trait that is difficult to trace on a phylogeny in itself [12,17,27], we propose that diverse host use within a taxon is a good indication of historical widening of the host plant range. It may be impossible to reconstruct which plant taxa were actual hosts at a given node in the phylogeny, but it is a fair assumption that an insect group using more plant taxa in total also has an evolutionary history involving more colonizations and hence more episodes with wider host plant ranges.

The most likely mechanism by which host expansions can increase the likelihood of speciation is that they allow the insect to gain a wider geographic distribution [50] and thus put it in a situation where genetic fragmentation is more likely. Obviously, geographic range expansions can also be caused by colonization of a single plant taxon with widespread distribution, such as the grasses, but on average the potential geographical range should expand when the host range increases.

The means by which plant-feeding influences diversification thus involve four interrelated processes: First, the host range increases through colonization of one or more novel host plants. Second, the wider host range allows the species to expand its geographical range and invade new habitats. Third, as polyphagy appears to be a relatively ephemeral phase evolutionarily [12], the interaction will probably eventually evolve towards specialization again, but populations in different parts of the geographical range can specialize on different parts of the combined host range, thus creating a geographic mosaic of more specialized populations [9,51].

Specialization can then promote genetic differentiation among populations either by assortative mating [10] or by increasing geographic fragmentation by allopatry [11]. Thus, plant-feeding insects may have reached their impressive species numbers not by a steady process of specialization and cospeciation, but by dynamic oscillations of host range.

This is a scenario that resembles the old biogeographical concepts of "taxon cycling" [52] or "taxon pulses" [53,54], where pulses of speciation are mediated through shifts between marginal and interior (such as island and mainland) habitats. Our scenario should be compatible with these models, even though we see no need for a stable 'center of diversification' around which the distributional ranges fluctuate. At this point, there is no indication of particular geographical regions that seem to be disproportionately represented in the diversification of nymphalid butterflies, but this question is certainly worth investigating more thoroughly [c.f. [55,56]]. Furthermore, as a wide host range often implies both behavioral and physiological plasticity, our scenario should also be compatible with the general hypothesis of diversification driven by phenotypic plasticity, as described by West-Eberhard [57].

We suggest that it is precisely because host specialization is *not *the "dead end" that it has often been interpreted as that plant-feeding insects have been able to become so species-rich. In fact, it is quite possible that the same applies to other diverse and often highly specialized groups, such as parasitoids and parasites. In the light of this, there is a need to direct more attention to the circumstances that can reverse the otherwise quite pervasive drive towards specialization seen in these groups.

## Conclusion

We show that diversity in host use within clades is a good predictor of species richness. As colonizations of novel plants are typically associated with host expansions such diversity is likely to have been caused by historical polyphagy [12]. Hence, we propose that much of the diversification of plant-feeding insects is driven by oscillations in host plant range, where host expansions allow the species to increase its geographical distribution and thereby setting the stage for subsequent population fragmentation by secondary specialization on different hosts in the repertoire. This latter stage can either be accomplished by allopatric isolation through increased geographic fragmentation [11] or by assortative mating [10].

## Methods

### Phylogeny

**Figure 4:**
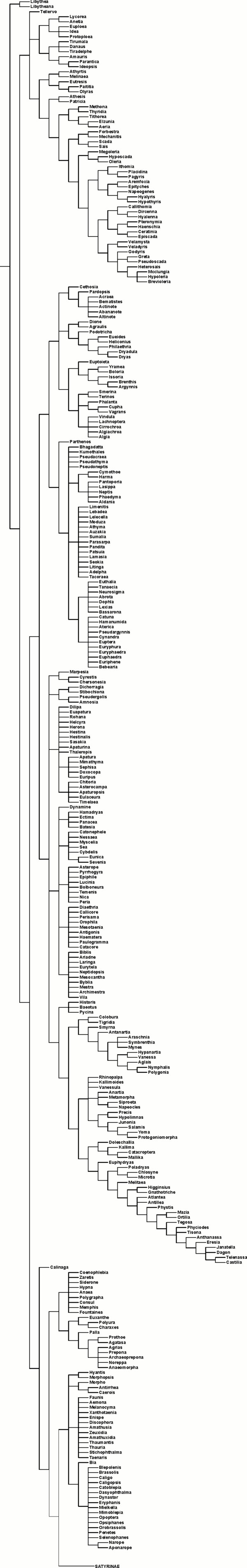
**Phylogeny of the Nymphalidae**. The phylogeny was compiled from several sources, using both molecular and morphological data (see Material and Methods for further information on the phylogenetic reconstruction and the sources used). It is resolved to the genus level, with the exception for the subfamily Satyrinae, which, due to little variation in host use, were counted as one contrast in the analysis.

A comprehensive phylogenetic analysis of all 548 genera belonging to Nymphalidae has yet to be published. However, the relationships of various subgroups in Nymphalidae have been studied by a number of people using diverse methodology, including morphological and molecular data. The phylogeny used in this study was compiled from various sources and represents our current understanding of the phylogeny of 309 genera of Nymphalidae, not including the subfamily Satyrinae (Fig. 4). The subfamily Satyrinae contains 239 genera and about 1/3 of all nymphalid species, but was treated as a single terminal taxon in this study, as it shows little variation in host use. It is not possible at this point in time to construct a supermatrix for analysis, as the datasets used by various people are largely non-overlapping in both characters and taxa. A supertree approach is not advisable based on recent work [58]. Thus we constructed the tree for Nymphalidae by taking the relationships of various clades directly from studies that focused on those particular relationships, as detailed below. The tree in Fig. 4 represents our current best estimate of Nymphalidae phylogeny, and all sister groups in our analyses are well-supported in the original publications dealing with their relationships.

Uncertainties in relationships are shown as polytomies in Fig. 4. The deeper nodes of the phylogeny are based on molecular data from 3 gene regions, the mitochondrial COI and the nuclear EF-1α and wingless, for a total of 2929 bp [59]. The study by Wahlberg et al. [59] identified 4 major clades in Nymphalidae: the danaine clade which includes the subfamily Danainae; the satyrine clade including Calinaginae, Charaxinae, Satyrinae and Morphinae; the heliconiiine clade including Heliconiinae and Limenitidinae; and the nymphaline clade including Nymphalinae, Cyrestinae, Biblidinae and Apaturinae. These major clades are also recovered in a broader study on butterflies and skippers [60], which also shows that the libytheines belong in the family Nymphalidae. Relationships of species in the subfamily Libytheinae have been studied using morphological data showing that the genera *Libythea *and *Libytheana *are monophyletic and each others sister groups [61].

Relationships with Danainae have been extensively studied using both morphological and molecular data. Species in the tribe Danaini (the milkweed butterflies) are the subject of a book [62] in which detailed morphological data were cladistically analyzed. All genera were found to form monophyletic entities. Relationships within the species rich tribe Ithomiini have been studied using morphological [63] and molecular [64] data. The molecular analysis was based on the same genes mentioned above.

Relationships within the satyrine clade have been poorly studied, even though the clade contains the most species in Nymphalidae. The relationships of *Morpho *and related genera are taken from a morphological study [65] and a molecular study based on one gene, wingless [66]. The position of *Bia *as sister to the rest of Brassolini is suggested by a morphological study [67] and is being confirmed by a molecular study based on COI, EF-1α and wingless genes [68]. The genera *Charaxes *and *Polyura *have always been considered to be related to each other [69] and this relationship is being confirmed with molecular data from the three genes mentioned previously (N. Wahlberg, unpublished data).

Relationships within the heliconiine clade have been much studied, especially in the subfamily Heliconiinae. These studies are based on both morphological [70-72] and molecular [73,74] data, and one study [75] combined both kinds of data.

Relationships in Limenitidinae have been less studied [76], and thus the phylogeny in Fig. 4 is largely unresolved for the subfamily. The sister relationship of *Harma *and *Cymothoe *is considered to be clear [77,78] and is being confirmed by molecular data from COI, EF-1α and wingless (N. Wahlberg, unpublished).

Relationships in the nymphaline clade are being cleared up presently. A very recent study on the subfamily Nymphalinae based on molecular data from COI, EF-1α and wingless [79] has resolved the phylogenetic relationships of almost all genera (hence the strong representation of this subfamily in the current study). Detailed studies on several subgroups within Nymphalinae have also been used in Fig. 4, the tribe Nymphalini was studied using a combined morphological and molecular dataset with four genes [80]. The other subfamilies in the nymphaline clade have not been studied in detail. The sister relationship of *Cyrestis *and *Chersonesia *was established in a morphological study [81] and is being confirmed by molecular data [79]. A few studies have used morphological data in the subfamily Biblidinae to look at relationships at the species level [82,83].

### Data collection

Data on host plant associations were collected from various literature sources on the level of plant family and order [62,67,77,78,84-101], following the nomenclature of the Angiosperm Phylogeny Group [102]. An index of host diversity was created by multiplying the number of plant families with the number of plant orders used by each butterfly genus. This allowed us to take variation into account on two levels of resolution, i.e. a butterfly genus utilizing two families in the same order will have a lower host diversity than a butterfly utilizing two families from two different orders. Butterfly species numbers were taken from various sources as detailed in Wahlberg [103]. We were able to find data on host plant use as well as species numbers for 292 genera in the family Nymphalidae. While butterflies are unparalleled among insects in terms of availability of host plant data, much due to the great general interest this group holds by amateur collectors as well as the general public, there are sometimes problems with the reliability of the data. The level of detail in host records vary substantially, which makes it difficult to use more fine-grained measures of host range than plant families. The largest problem is probably that anecdotal and erroneous records will tend to spread and multiply in the literature, and these can decrease the phylogenetic signal in the data [27,84]. To limit this problem, we have followed a set of evaluation rules adopted from Janz & Nylin [27], where a host plant association was only included if it was a) reported by at least two independent sources, b) recorded from more than one species in the genus, c) if there were records of more than one plant genus from the same host plant family, or d) if the plant was the only recorded host for the genus. Because of the way that atypical information tends to gain undue attention, we believe that the risk we hereby run of erroneously excluding some data that are correct is outweighed by the advantage of excluding a greater number of records that are incorrect.

### Analyses

Data were analyzed with the MacroCAIC computer program [45], which applies the method of independent contrasts [104] to questions of species richness, as well as by reciprocal sister-group comparisons. MacroCAIC generates phylogenetically independent contrasts across the whole phylogeny. By taking all available data into account the number of contrasts found will be much higher than when manually searching for valid sister group comparisons, but by using reciprocal selection rules for sister group selection it is possible to evaluate the causal relationships behind a correlation.

As our data were strongly skewed (most butterflies are specialists) we were not able to use a parametric regression on the contrasts generated by MacroCAIC. Instead we performed a Wilcoxon matched-pair signed-rank test on the direction of the contrasts, which is also more conservative and more comparable with the sister group comparisons. This test answers the question of whether positive contrasts in one variable (diversity of host use) are associated with positive contrasts in the other variable (species richness).

Sister groups were selected using two reciprocal selection rules (Fig. 1). First, by recursively searching down the butterfly phylogeny for the first sister pair that differed in diversity of host use. At that point we stopped, excluding all nodes below it in the phylogeny (so as to ensure independent comparisons). We then continued in the same manner across the whole phylogeny until all possible independent sister groups were found (Table 1). Second, we used the same method to instead search for clades that differed in species numbers (Table 2). This reciprocity allowed us to evaluate our hypothesis against the alternative hypothesis that species rich clades are expected to have more hosts by chance alone. Valid comparisons of both types were analyzed with sign tests for finding correlated differences in host diversity and species richness. Provided that there is an overall correlation, in many (most) cases, the sister group contrasts found with the two methods will be identical. However, as long as the correlation is not perfect, there will be cases where a difference in one character is not followed by a difference in the other. If these discrepancies from a perfect correlation are disproportionately found in one of the sister group comparisons, they can provide insight into the causation behind the correlation. For example, we may find a large number of contrasts in species richness that is not associated with positive contrasts in host diversity, but few cases of the reciprocal discrepancy (where a contrast in host diversity is not associated with a positive contrast in species richness). This means that differences in species richness sometimes evolve for reasons not associated with host diversity, but when there is a difference in host diversity, it is predictably followed by an increase in species richness. Conversely, the opposite pattern would mean that host diversity often evolves without a corresponding increase in species richness, but when there are differences in species richness, it is predictably followed by an increase in host diversity. In the first case we would conclude that, even though host diversity cannot be the only factor that influences patterns of species richness, when we do have an increase in host diversity, it seems to trigger an increase in species richness. In the second case we would conclude that, even though species richness cannot be the only factor that influences patterns of host diversity, when we do have an increase in species richness, it seems to trigger an increase in host diversity.

## Authors' contributions

NJ and SN conceived of the study and made preliminary analyses, NJ collected the data, carried out final analyses and wrote the paper, NW performed the phylogenetic reconstructions and wrote part of the methods. All authors partook in discussions during analysis and writing, read and approved the final manuscript.
